# Attenuation of oxidative stress & DNA damage in varicocelectomy: Implications in infertility management

**Published:** 2010-12

**Authors:** Rima Dada, Monis Bilal Shamsi, Sunderarjan Venkatesh, Naramada Prasad Gupta, Rajeev Kumar

**Affiliations:** Department of Anatomy, All India Institute of Medical Sciences, New Delhi, India; * *Department of Urology, All India Institute of Medical Sciences, New Delhi, India*

**Keywords:** Infertility, oxidative stress, semen, varicocelectomy

## Abstract

Sperm DNA integrity is of vital importance for foetal development and birth of healthy offspring. Oxidative stress and consequent DNA damage are the major cause of decline in semen quality in men with varicocele. A preliminary study was conducted on 11 men with clinical varicocele who also had high levels of reactive oxygen species (ROS), to assess DNA damage in sperms and ROS levels before and after varicocelectomy. Varicocelectomy resulted in rapid (1 month) significant (*P*<0.001) decline in free radical levels and slow (3-6 months) significant decline in DNA damage levels. Thus men undergoing varicocelectomy should try concieving only 6 months following surgery.

Varicocele is one of the commonest surgically reversible causes of infertility and affects about 40 per cent males with primary infertility and 70-80 per cent males with secondary infertility. The pathophysiology of varicocele induced infertility remains unknown. One of the mechanisms by which it causes a decline in semen quality is increased testicular temperature due to dilation and tortuosity of pampiniform plexus of veins^1^. In addition, a number of men with varicocele harbour genetic abnormalities like Yq microdeletions^2^. However, it is unknown whether these are causally related or simply an incidental finding because of the high prevalence of varicocele among infertile men.

Supraphysiological reactive oxygen species (ROS) levels have been reported in men with varicocele and is one of the potential aetiological factors in varicocele mediated deterioration in sperm concentration, motility and morphology^3^,^5^. Agarwal *et al*^6^ reported that sperm dysfunction is multifactorial in varicocele but oxidative stress is the main cause. Oxidative stress can cause an alteration in the dynamics of testicular microvascular blood flow, endocrine signaling and germ cell apoptosis^7^.

We conducted a preliminary study at the All India Institute of Medical Sciences, New Delhi, from January 2009 to January 2010 on 11 men (age 31.09 ± 2.8 yr) with clinical varicocele (cytogenetically normal, no Yq microdeletion, non smokers, non alcoholic, normal basal metabolic index, no history of testicular or systemic infection), who came for follow up for 3 and 6 months post-varicocelectomy. All men had significantly high ROS levels in washed and neat semen (3,121,725.65 and 142,897.704 RLU per 20 million sperm/min respectively) as compared to fertile age matched healthy controls (n=15) (800-1800 and 400-800 RLU per 20 million sperm/min). The fertile controls were men attending the clinic for vasectomy. One month after varicocelectomy, there was a significant (*P*<0.001) decline in ROS levels to 159,001.838 and 10,776.736 RLU per 20 million sperm/min in washed and neat semen respectively. Three months post-varicocelectomy the ROS levels further reduced to 98,971.081 and 6,456.249 RLU per 20 million sperm/min in washed and neat semen respectively. ROS levels were measured by chemiluminiscence method using luminol as probe^8^. The study protocol was approved by the Institute Ethics committee. The statistical comparison was done by Student’s t test.

DNA damage in sperms was assessed before and after varicocelectomy (3 months) by comet assay^9^ (single cell gel electrophoresis) in all 11 patients and showed no significant difference in DNA damage levels at 3 months post-varicocelectomy (despite significant decline in ROS levels). Pre-varicocelectomy the mean DNA content in comet head was 39.18 ± 7.12 per cent which showed non-significant improvement to 41.21 ± 5.21 per cent one month after varicocelectomy and 44.02 ± 6.12 per cent three months post-varicocelectomy (quantity of DNA in comet head is directly proportional to DNA integrity).

Six of the 11 men came for follow up after 6 months of varicocelectomy. The ROS levels had declined further to 45,234.926 and 4,528.593 RLU per 20 million sperm/min in washed and neat semen respectively. At this point, these patients showed a significant improvement in the sperm DNA integrity and the mean DNA content in the comet head increased to 67.42 ± 6.12 per cent, as compared to 44.02 ± 2.54 per cent at three months post-varicocelectomy.

Oxidative stress is a major cause of both nuclear and mitochondrial DNA damage^8^, and thus decline in ROS levels may also lead to decrease in DNA damage. DNA integrity improved significantly only 6 months after surgery. This may be because the spermatogenic cycle lasts for 64-72 days and at least one full cycle will need to be completed before the beneficial effects are evident. This finding is of clinical significance as men with varicocele may be counselled to try conceiving approximately 6 months post varicocelectomy.

Oxidative stress also induces mitochondrial DNA mutations, nuclear DNA breaks/nicks and nucleotide modification and thus reduces sperm functional competence by altering its membrane permeability and fluidity^9^,^10^. Sperm oxidative stress also causes pronuclear block, slows cleavage and sperm DNA damage, leads to pre- and post-implantation failure, increased risk of major and minor congenital malformations, genetic and epigenetic defects and cancer in off spring^11^,^13^.

Oxidative stress and oxidative stress induced DNA damage appear to be major factors which adversely affect semen quality and lead to infertility. Use of such sperm for ART may adversely affect fertilization, embryogenesis and may have lifelong implications on foetal health. Results of this pilot study highlight that infertile men should be counselled to try conceiving only 6 months following varicocelectomy. Varicocelectomy may be beneficial in reducing the ROS levels, oxidative stress induced DNA damage with an overall improvement of functional competence of sperm.
